# Dermatofibrosarcoma protuberans challenges: a case series and review of the literature 

**DOI:** 10.1186/s13256-022-03728-6

**Published:** 2023-01-19

**Authors:** Somayeh Sheidaei, Mahsa Salehi, Fatemeh Abedian kenari, Hamid Reza Jafari

**Affiliations:** 1 Pathology, Mazandaran University of Medical Sciences, Mazandaran, Iran; 2 General surgery, Isfahan University of Medical Sciences, Isfahan, Iran

**Keywords:** Dermatofibrosarcoma protuberans (DFSP), Wide local excision (WLE)

## Abstract

**Background:**

Dermatofibrosarcoma protuberans (DFSP) is a rare variant of skin sarcoma which is characterized by proliferation of spindle cells in a storiform pattern. Although it is mostly benign in its primary stages, it can cause a high burden of morbidity unless it is thoroughly excised.

**Case presentation:**

Here, we review six cases of DFSP which were characterized by skin lesions in various parts of the body. Patients were from 26 to 51 years old; four were Asian men and two were Asian women. Wide surgical excision was performed for all these patients and no extra treatment was considered. Samples were studied by hematoxylin and eosin (H&E) staining and immunohistochemical (IHC) tests. Only one of our patients experienced recurrence after the initial surgery.

**Conclusion:**

Determining the best surgical method is still a dilemma in the treatment of DFSP lesions. There are numerous studies to prove the efficacy of various surgical interventions. Although DFSP is not commonly known as a malignant skin lesion, delay in treatment will have a catastrophic impact on patients’ lives. Thus, applying an in-time surgical method (wide local excision in our cases) in treating DFSP is crucial in preventing recurrence as well as decreasing the morbidity burden of DFSP.

## Background

Dermatofibrosarcoma protuberans (DFSP) is a rare dermal and soft tissue tumor which originates from dermal fibroblasts. In clinical terms, DFSP is a low-grade and slow-growing form of sarcoma occurring mostly in middle-aged individuals, with a higher prevalence in females [1–3]. Histologically, DFSP consists uniformly of spindle cell fascicles positioned in a storiform pattern. However, due to various CD34 immunoreactivity in DFSP, its presentation can be mistaken with other malignancies or even benign lesions [4–7]. The majority of DFSP cases have a t (17; 22) (q22; q13) which results in the creation of COL1A1-PDGFB fusion transcripts [8–11]. Although it has low metastasis risk, it has significant destructive effects locally, which is mostly diagnosed in lesions of the trunk area and distal part of the extremities [12]. Some cases of DFSP in the head and neck region are reported, as well [1].

With regard to the morphological appearance of DFSP, it is a firm, painless, and pink or erythematous dermal nodule in its early stages similar to other types of atrophic benign skin lesions. Consequently, confirmed diagnosis by physical examination can be a challenge. Therefore, diagnosis of DFSP is achieved by histopathological and immunohistochemical (IHC) tests [12, 13]. Generally, magnetic resonance imaging (MRI) and fluorescence *in situ* hybridization (FISH) along with reverse transcriptase polymerase chain reaction (RT-PCR) can be utilized not only for the screening but also for the purpose of choosing the best treatment.

DFSP is treated primarily by surgical intervention in the majority of cases. It is advised that complete resection be achieved in the first attempt due to the high risk of recurrence. Furthermore, the risk of metastasis seems to increase in incomplete resections and repeated surgical interventions in DFSP [1, 14]. Mohs micrographic surgery (MMS) and wide local excision (WLE) are surgical options for the treatment of DFSP. However, there are conflicting results in terms of choosing the most suitable type of surgical treatment for DFSP patients [3, 8, 10–15]. In the following study, we report our own experience of diagnosis and treatment of six cases of DFSP and review recent updates in the journey toward finding the best treatment for patients with DFSP.

## Case presentation

In our case study, we received specimens from patients between 2014 and 2019 in one hospital. These samples were provided by one surgeon and were examined by hematoxylin and eosin (H&E) staining and IHC tests. Out of these samples, six specimens were diagnosed with DFSP by CD34-positive marker in IHC tests besides other markers. Technically, WLE was considered for all these patients. Four of the patients underwent biopsy before the surgical excision. Written informed consent was obtained from the patients for publication of this case report and any accompanying images.

**Fig. 1:**
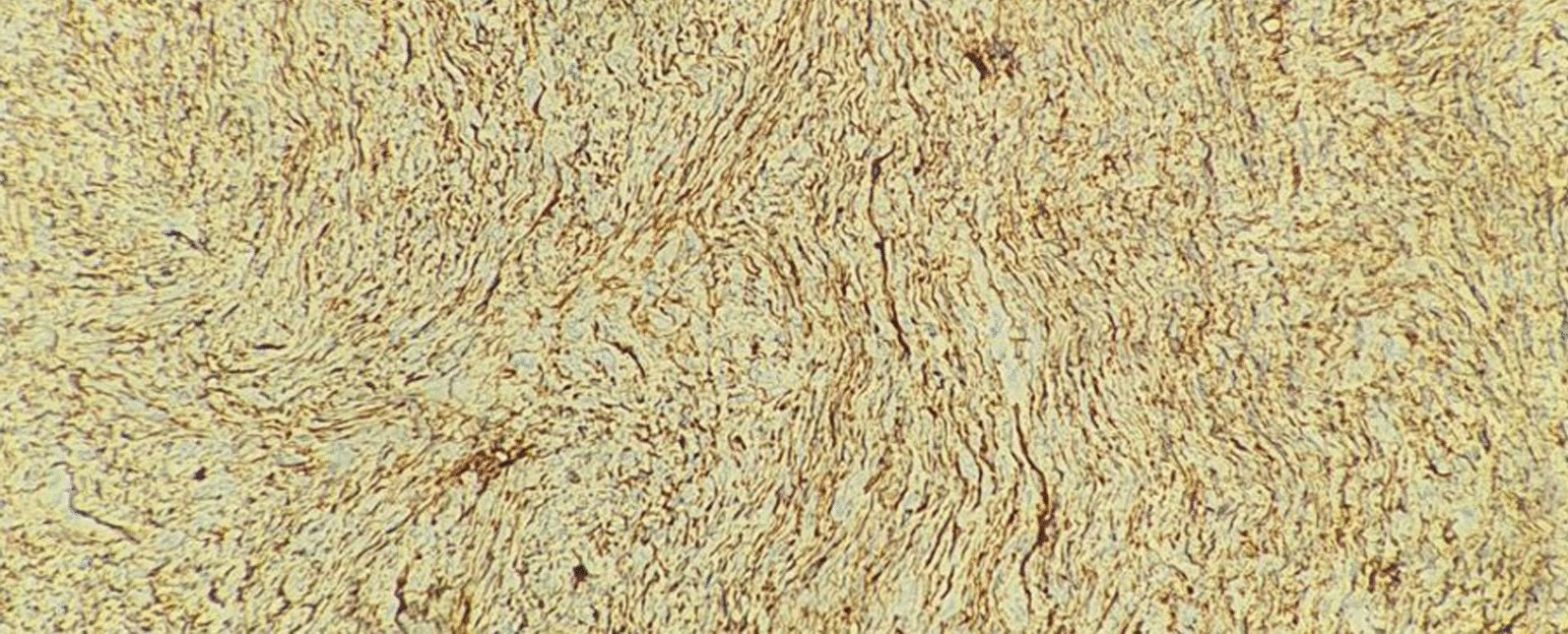
IHC panel for CD 34

**Fig. 2:**
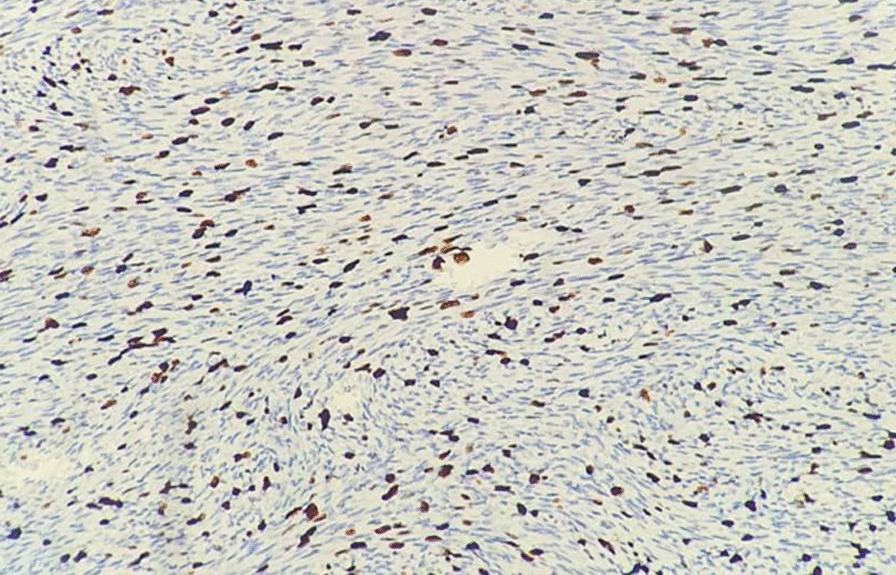
Ki67 testing of samples

Turning to the data of our patients in Table 1, four patients were male and two were female. Their ages ranged from 26 to 51 years, with a mean age of 32.5 years. Considering the location of the lesions, three were excised from the trunk, one from the scrotum, and two samples from the limbs. The lesions ranged in size from 1.6 cm to 7 cm. Margins were from 1.9 to 3.1 cm, with a mean of 2.4 cm. In our IHC panel, we tested for CD34 (Fig. 1), smooth muscle actin (SMA), and Ki67 (Fig. 2). CD34 was positive in all of our cases, in contrast to negative SMA staining. Ki67 was between 5 and 10%.

**Fig. 3:**
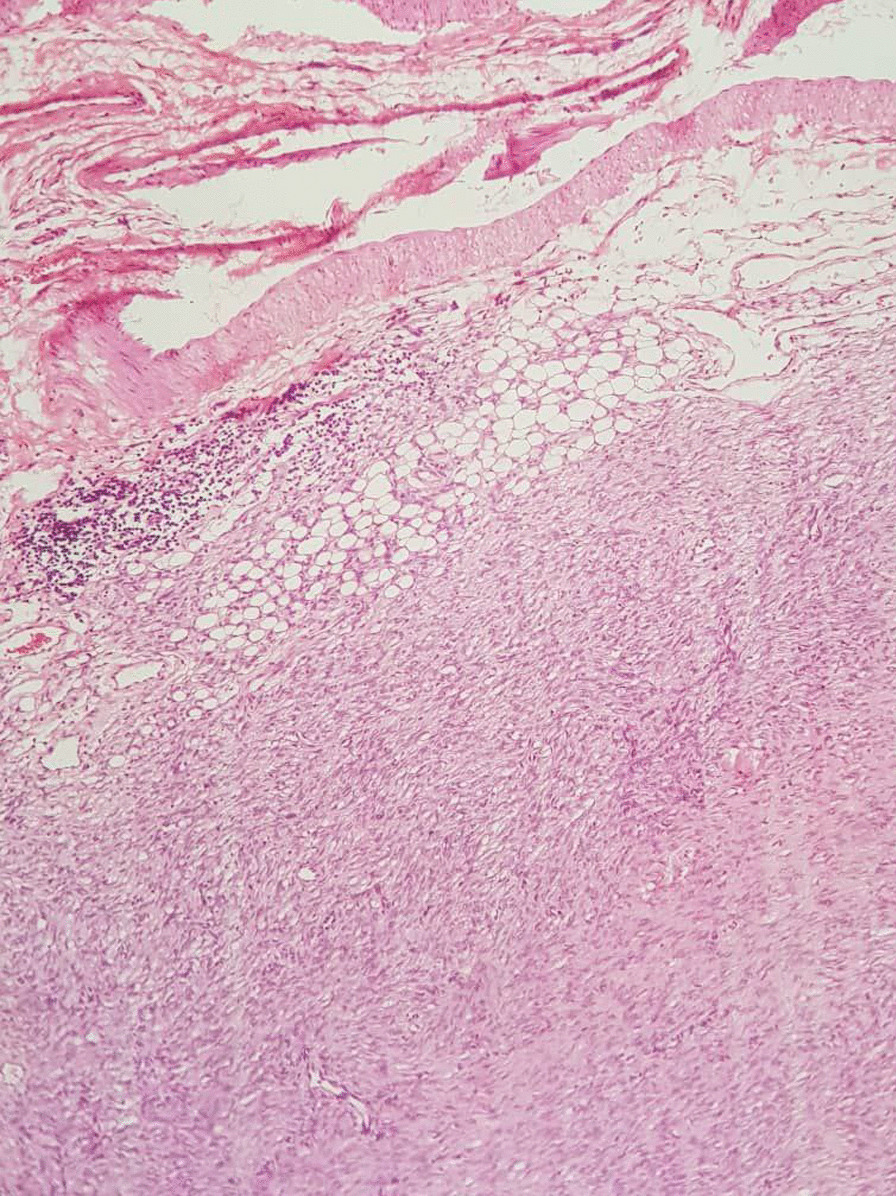
Sections of ill-defined mesenchymal neoplasm involving papillary and reticular dermis

**Fig. 4:**
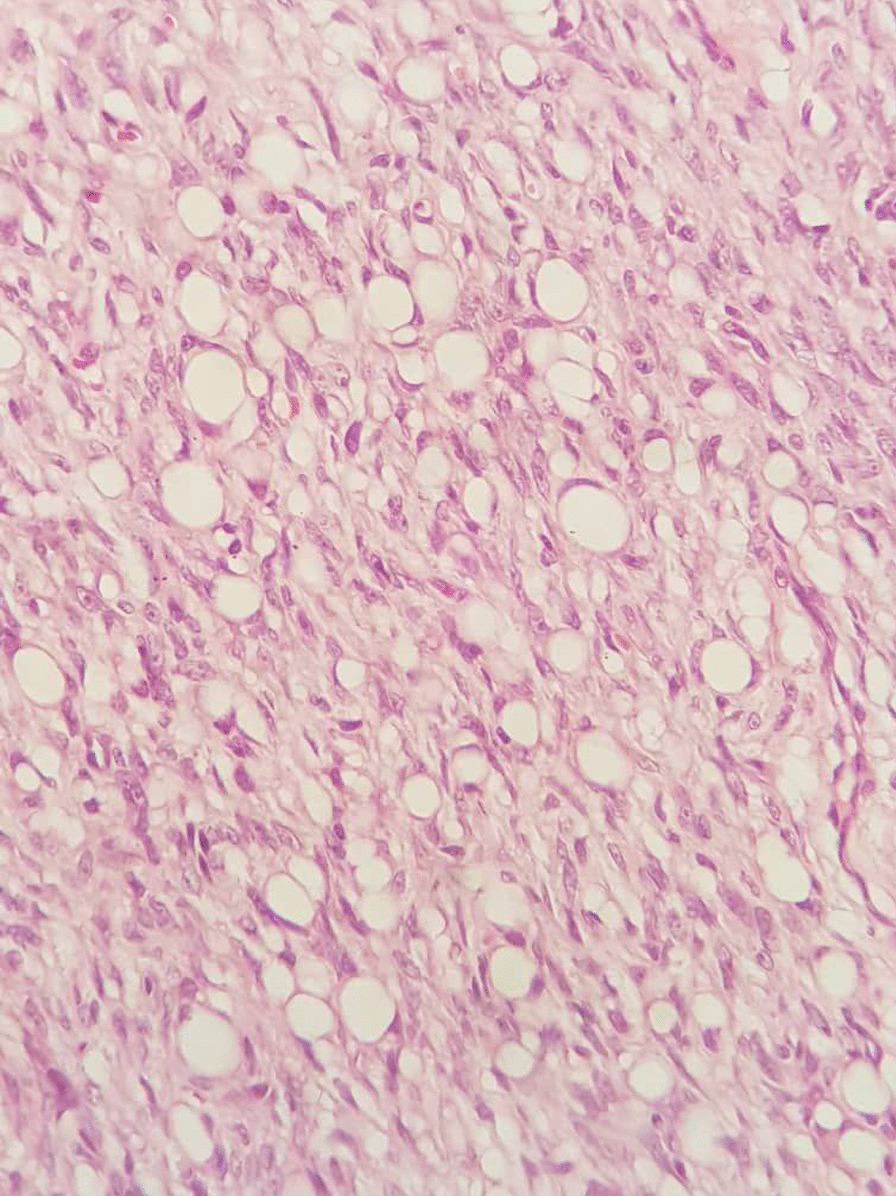
Sections of ill-defined mesenchymal neoplasm involving papillary and reticular dermis

**Fig. 5:**
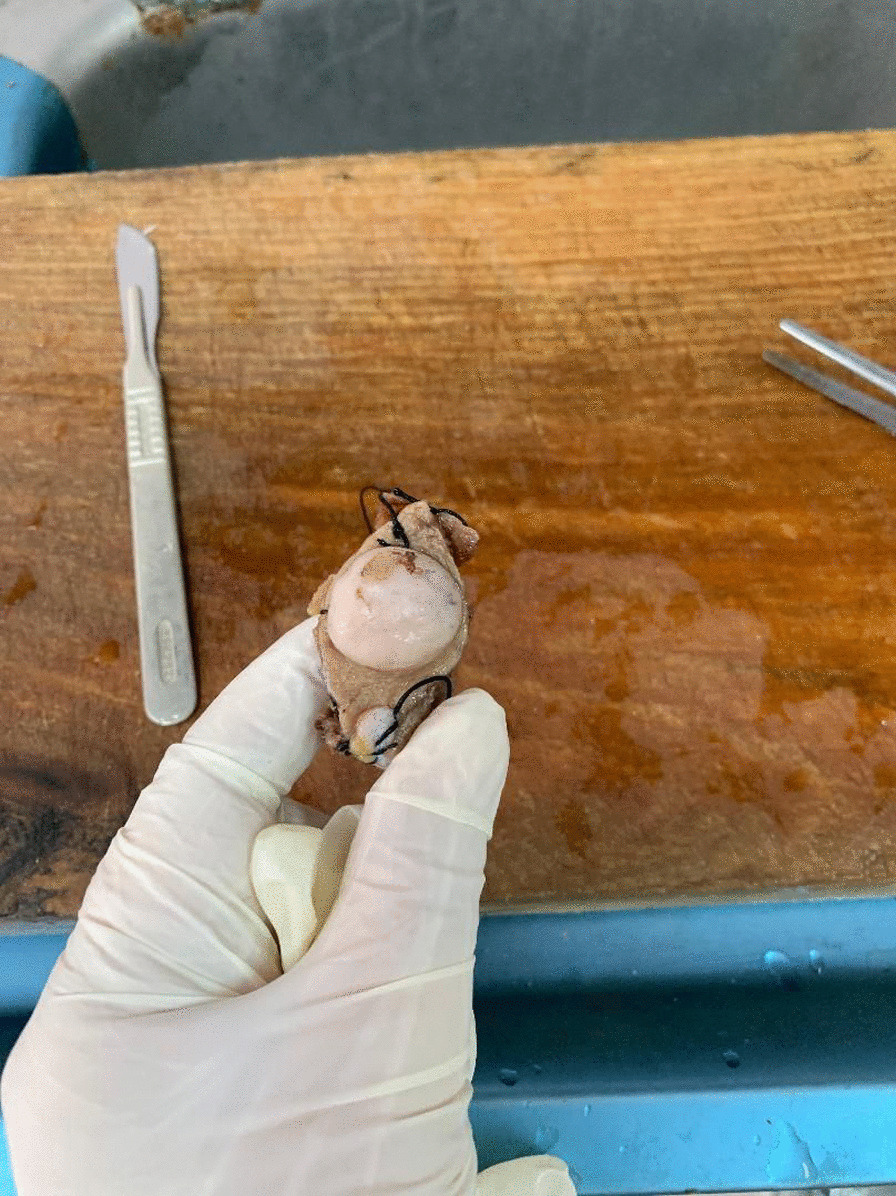
Atrophic variation of DFSP lesion

**Fig. 6:**
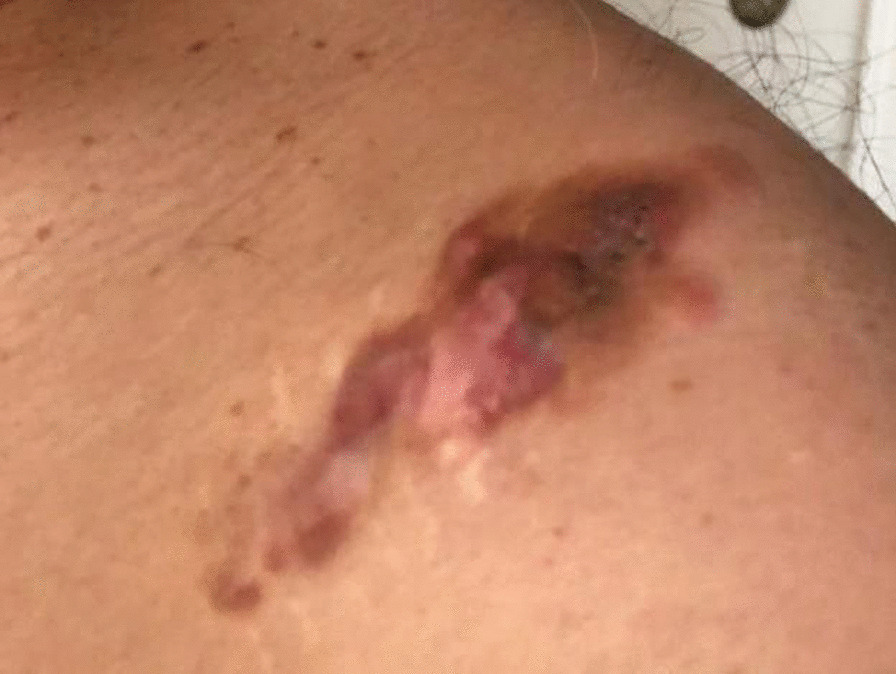
Bednar tumor

Based on the staging table provided by one study [1], 50% of our case were in stage IIA, one in stage IIB, and two in stage I. Regarding the pathology study of our specimens, sections revealed thin skin epidermis with ill-defined mesenchymal neoplasm involving papillary and reticular dermis (Figs. 3, 4) The lesions were composed of a uniform population of slender fibroblasts arranged in a storiform pattern around in conspicuous vasculature and dermal appendages. Nuclear pleomorphism was absent and mitosis was rare. Melanin-containing cells were scattered throughout the lesions. At the deep dermis, entrapment of fatty tissue parallel to the surface was also seen. Four of the cases were diagnosed with atrophic lesions (Fig. 5) and two with Bednar tumors (Fig. 6) Shoulder MRI of one of our patients revealed a well-defined homogeneous isointense lesion. One chest computed tomography (CT) study showed an enhanced solitary nodule, while ultrasonography of the scrotum indicated a superficial nodule.

Our follow-up lasted from 11 to 81 months. One of our patients being in stage IIB, who had the largest lesion, experienced recurrence, while the rest had no further problems. He underwent re-resection due to a positive margin 2 weeks after his primary surgery. However, during his 61 months of follow-up, there was no evidence of DFSP relapse. Half of our patients required reconstruction in their surgical sites. No patients had neoadjuvant theory or radiotherapy before or after their surgeries.

## Discussion

### Clinical features

DFSP is a rare skin tumor which was first described in 1924 as an invasive and progressive dermatofibroma. Its annual incidence is as low as one case per million population. Although recent studies have shown no gender preference in the prevalence of DFSP, it is known to be most common in middle-aged male patients. Although it shows a benign pattern in the early stages, it has the potential to turn into a high-grade fibrosarcoma on rare occasions. Moreover, years after the initial diagnosis, a minority of nearly 6% can invade distant organs such as the lungs [16]. However, one study reported 15% lung metastasis in DFSP cases in correlation with other forms of high-grade sarcomas [6, 13, 17–19]. Age and gender are not risk factors for DFSP and its recurrence. The trunk and extremities are the most common locations for DFSP, while recent studies have shown that about 40% of lesions were found in the head and neck region. Moreover, the recurrence rate of head and neck lesions was higher in some surveys, albeit lacking statistical difference [6, 13].

Since DFSP lesions are great masqueraders as benign skin lesions, they can resemble masses such as lipoma, cyst, or even scars which are removed for cosmetic purposes. In light of this information, it is clinically reasonable to obtain biopsy or choose a wide local excision approach for lesions which are cystic in appearance but lack a punctum or malodorous pus-like secretions. Moreover, surgeons should have second thoughts when they want to remove a skin mass larger than 2.5 cm or that is increasing in size and lesions with alteration in their physical appearance. Also, pain can be a warning sign of malignancy in DFSP lesions. It is advised that such patients should be referred to a cancer center with staff members experienced in cases of lesions with suspected sarcoma, for the purpose not only of preoperative microscopic study of the lesions, but also their treatment [8, 18, 20]. As the DFSP grows to late stages, it can be ulcerative, painful, and bleeding, in contrast to a low-grade lesion [16].

In the early stages of DFSP, they are non-protuberant lesions, turning into indurated, purple, and violaceous nodules as they grow. Left unresected, tumor regions can reach the underlying tissue such as fascia, muscle, periosteum, and bone. It can even cause distal metastatic lesions in organs such as the lungs, brain, and lymph nodes. However, this process can range from a few months to decades [1, 19, 20]. DFSP lesions shows various forms. The atrophic and pigmented forms are rare variants. The pigmented DFSP lesions, also called Bednar tumors, usually have the same color as a bruise of dark blue or black appearance. Such lesions have melanin-containing dendritic cells besides other histological findings of DFSP. On the other hand, the atrophic variant is mostly a flat plaque rather than the usual protuberant lesions, resembling a dermis-based skin mass with typical plaque-like histological features beside dermis layer thinning. However, these two variants have spindle cells with vimentin and positive CD34 staining. Gender has no impact on the aforementioned variants. Furthermore, they are mostly removed from the trunk and extremities. Contrary to the usual findings, atrophic pigmented lesions can occur in children as well [14, 21, 22]. Although there is no accepted staging system among all these studies, one study provided a table based on clinical features of DFSP lesions [1].

### Diagnosis

The differential diagnosis for DFSP lesions is dermatofibroma, schwannoma, cutaneous neurofibroma, solitary fibrous tumor, intradermal spindle cell lipoma, and spindle cell or desmoplastic melanoma [1]. DFSP cases are usually an unexpected pathological result of a presumably benign skin mass. Thus, it can be challenging when a safe margin was ignored in such patients [2, 5, 15, 17, 23]. Such conditions can result in a 3- to 5-year delay in diagnosis of patients with cases suspicious for DFSP [13]. With regard to diagnosis, CD34 marker in IHC investigation of samples by sensitivity of more than 85% has a key role in differentiating DFSP from other benign soft tissue tumors [16].

MRI can function as a diagnostic tool in measuring the DFSP tumor size and their invasion of neuromuscular tissues and bone. With respect to findings, MR T1-weighted images represent well-defined homogeneous isointense lesions as well as exhibiting a well-defined subcutaneous soft tissue lesion by an intermediate to marked homogeneous hyperintensity to the surrounding muscular area. Ultrasound can reveal a hypoechoic superficial nodular lesion. Furthermore, CT studies with contrast demonstrate enhanced solitary, subcutaneous lobular or nodular lesions with soft-tissue attenuation. CT scans not only can identify distant metastasis in DFSP patients, but also can reveal necrosis or cystic degeneration in dark areas of tumors larger than 5 cm [1, 3, 24].

### Surgical treatment

In terms of treatment of DFSP, complete surgical excision is the gold standard [16, 25]. Nonetheless, neoadjuvant therapy with imatinib and radiotherapy is suggested in the majority of cases that experienced incomplete resection. Such cases suffer from recurrence as well. Indeed, the margin of surgical resection was found to have a direct impact on the risk of future recurrence. The most crucial prognostic factor is the extent of surgical excision, which indicates the importance of removal of deeper involved tissue such as deep fascia and muscles. Failure to make a through excision of the lesion could result in local invasion due to its infiltrative behavior. The recurrence rate varies from 10 to 80% in studies. However, one study reported that the recurrence was double in lesions with margins less than 2 cm in comparison with those with a larger diameter [16, 19].

Various studies have focused on Mohs and WLE as the commonly accepted type of surgery. Traditionally, wide margins of 2 to 4 cm with careful pathological study of the resection line will usually prevent future recurrence in DFSP tumors [26]. There are conflicting results in considering the width of the surgical margin, since there is no universal recommendation for the diameter of resection in WLE as in British Sarcoma Group guidelines. On the other hand, National Comprehensive Cancer Network (NCCN) guidelines consider 2–4 cm a safe margin for DFSP [17]. Such approaches could end in higher morbidity as well as requirement for further surgical reconstructions by roughly 50% [4, 17]. Hence, an alternative approach of conservative re-excision (CRE) by narrower margins was investigated by one study. In the CRE method, 1.5 cm is considered the mean of excision margin for DFSP cases. CRE, especially in areas such as the scalp and thigh, can reduce the probability of longer operation time and hospitalization in addition to controlling the need for normal skin for reconstruction of surgical sites by approximately 7%. Even though both WLE and CRE are accepted in oncological settings, there is the dilemma of determining whether to initiate WLE with a higher chance of reconstruction, or a CRE procedure with lower morbidity and risk of positive margin and further reoperation [2, 10, 11, 17, 27]. Frozen section has also been used to examine the margins during DFSP surgeries to prevent further need for re-excision. Meanwhile, it can provide false-positive results for DFSP due to fibroblastic proliferation in correlation with the scar tissue of previous excisions. Also, CD34 IHC tests, as a consistent test for DFSP diagnosis, cannot be utilized during frozen section for assessing intraoperative margins [17]. Out of almost 200 DFSP patients, the majority of patients underwent WLE excision, with a scar larger than 10 cm in the end, in contrast to conventional excision (CE) resection. In micrographic surgery, the scar size of WLE and MOHS was similar. To further elaborate, the goal in the CE method is to remove large tumors from sensitive areas such as the groin and face with minimal scar instead of providing a negative margin. Thus, it is inevitable that the recurrence rate in CE patients was almost six times higher than the WLE and Mohs techniques [8].

Although the MOHS method can play a major role in reducing the rate of recurrence, it is highly reliant on the capabilities of surgeons and pathologists as well as time-consuming and costly. Considering other downsides of MOHS, spindle cells of the normal dermis can be mistaken for tumoral cells in frozen sections. Furthermore, CD34 staining can yield inconsistent results or even false data. Therefore, it can raise doubts regarding the reliability of the MOHS technique despite its benefits [13, 20], and WLE is a more preferred technique in clinical practice. The recommended margin for resection of DFSP tumors is 2 cm in general. The aim is to make the distance between tumor edge and cutting edge more than 1 cm. In one study of 260 patients with DFSP, the authors reported a significant difference between patients with a margin of 1.5–2.5 cm and those above 3 cm [13]. The recurrence rate with Mohs and WLE methods showed conflicting results. Generally, Mohs surgery of DFSP lesions had a lower recurrence rate of 3% compared with WLE; however, some recent studies reported 5% recurrence for the Mohs procedure [8].

Generally, WLE with safe margins should be considered for the majority of DFSP cases of the trunk and extremities. The Mohs method is suggested for sensitive areas to preserve tissue for cosmetic purposes as well as saving organ function. Meanwhile, amputation or adjuvant targeted radiotherapy is considered when surgical removal is out of reach. Follow-up evaluation every 6 months is recommended for a follow-up period of 5 years after surgical intervention [1].

### Nonsurgical treatment

There are inconsistent outcomes regarding the importance of using post-operation radiotherapy. Some meta-analytical data showed that the impact of neoadjuvant therapy on the recurrence rate was not significant in comparison with surgical resection alone. Moreover, radiation after surgery can cause skin flap necrosis, edema, and delayed healing in the resection site. Hence, the choice of radiotherapy is highly dependent on the patients’ conditions [9, 13]. Some studies have suggested the use of traditional post-op radiotherapy since DFSP is known to be radiosensitive. In fact, radiotherapy can be life-saving in tumors with questionable margins or inoperable lesions to reduce the morbidity, distant metastasis, and need for extensive reoperation [15, 16]. Regarding immunotherapy, in addition to imatinib, sorafenib is successful in the treatment of angiosarcomas. It inhibits vascular endothelial growth factor (VEGF) and was used to treat a case of refractory DFSP unresponsive to postoperative radiation [2, 3, 10, 16, 27].

## Conclusion

DFSP is a rare but challenging skin lesion. It can cause morbidity in patients in the absence of proper surgical treatment. Although new microsurgical methods can be useful in the treatment of DFSP, the classic WLE can be considered the choice of treatment in the majority of cases. However, further clinical trial studies are needed to truly compare the end result of various types of surgical interventions in DFSP. Moreover, regular long-term follow-up is vital in managing this type of skin mass.

**Table 1:** The table summerizes demographic, clinical, and pathological data of patients

Patient	Age (years)	Gender	Location of the lesion	Pre-op biopsy	Type of surgery	Size (cm)	Margin (cm)	Positive margin	Variant	Imaging	IHC panel			Stage	Post-op reconstruction	Follow-up period (months)	recurrence	Neoadjuvant therapy
											CD34	SMA	Ki67					
A	51	Male	Scrotum	Yes	WLE	2.5	2	No	Atrophic	Ultrasonography	Positive	Negative	<5	I	No	39	No	No
B	29	Male	Upper limb(shoulder)	Yes	WLE	7	2.5	Yes	Pigmented (Bednar tumor)	MRI	Positive	Negative	<10	IIB	Yes	61	Yes	No
C	26	Female	Lower limb(thigh)	No	WLE	1.7	2.2	No	Atrophic	None	Positive	Negative	<10	IIA	No	68	No	No
D	38	Male	Trunk(chest)	Yes	WLE	3	3.1	No	Pigmented (Bednar tumor)	CT scan	Positive	Negative	<5	IIA	Yes	11	No	No
E	35	Female	Trunk(back)	Yes	WLE	2.8	1.9	No	Atrophic	None	Positive	Negative	<5	IIA	No	16	No	No
F	46	Male	Trunk(back)	No	WLE	1.6	2.8	No	Atrophic	None	Positive	Negative	<10	I	Yes	81	No	No

*CT* computed tomography, *IHC* immunohistochemical, *MRI* magnetic resonance imaging, *WLE* wide local excision

## Data Availability

Not applicable.
